# Collaboration between emergency physicians and citizen responders in out-of-hospital cardiac arrest resuscitation

**DOI:** 10.1186/s13049-021-00927-w

**Published:** 2021-08-03

**Authors:** Anne-Sofie Linde Jellestad, Fredrik Folke, Rune Molin, Rasmus Meyer Lyngby, Carolina Malta Hansen, Linn Andelius

**Affiliations:** 1 Copenhagen Emergency Medical Services, University of Copenhagen, Telegrafvej 5, opgang 2, 3. sal, 2750 Ballerup, Denmark; 2 Department of Clinical Medicine, University of Copenhagen, Copenhagen, Denmark; 3 Department of Cardiology, Herlev-Gentofte University Hospital, Copenhagen, Denmark; 4 Kingston University and St. Georges, University of London, London, UK

**Keywords:** OHCA, EMS, First responders, CPR, Bystander, Dispatch

## Abstract

**Background:**

Citizen responder programmes dispatch volunteer citizens to initiate resuscitation in nearby out-of-hospital cardiac arrests (OHCA) before the Emergency Medical Services (EMS) arrival. Little is known about the interaction between citizen responders and EMS personnel during the resuscitation attempt. In the Capital Region of Denmark, emergency physicians are dispatched to all suspected OHCAs. The aim of this study was to evaluate how emergency physicians perceived the collaboration with citizen responders during resuscitation attempts.

**Method:**

This cross-sectional study was conducted through an online questionnaire. It included all 65 emergency physicians at Copenhagen EMS between June 9 and December 13, 2019 (catchment area 1.8 million). The questionnaire examined how emergency physicians perceived the interaction with citizen responders at the scene of OHCA (use of citizen responders before and after EMS arrival, citizen responders’ skills in cardiopulmonary resuscitation (CPR), and challenges in this setting).

**Results:**

The response rate was 87.7% (57/65). Nearly all emergency physicians (93.0%) had interacted with a citizen responder at least once. Of those 92.5%(*n* = 49) considered it relevant to activate citizen responders to OHCA resuscitation, and 67.9%(*n* = 36) reported the collaboration as helpful. When citizen responders arrived before EMS, 75.5%(*n* = 40) of the physicians continued to use citizen responders to assist with CPR or to carry equipment. Most (84.9%, *n* = 45) stated that citizen responders had the necessary skills to perform CPR. Challenges in the collaboration were described by 20.7%(*n* = 11) of the emergency physicians and included citizen responders being mistaken for relatives, time-consuming communication, or crowding problems during resuscitation.

**Conclusion:**

Emergency physicians perceived the collaboration with citizen responders as valuable, not only for delivery of CPR, but were also considered an extra helpful resource providing non-CPR related tasks such as directing the EMS to the arrest location, carrying equipment and taking care of relatives.

**Supplementary Information:**

The online version contains supplementary material available at 10.1186/s13049-021-00927-w.

## Introduction

Citizen responder programmes have been implemented in many communities to increase bystander cardiopulmonary resuscitation (CPR) and defibrillation before the arrival of the Emergency Medical Services (EMS) [1–5]. Activation of volunteer citizens through a smartphone application or text messages has been associated with increased likelihood of receiving bystander CPR and shorter time to defibrillation compared with situations where EMS arrive at the OHCA location first [5–10].

A citizen responder programme was implemented in September 2017 in the Capital Region of Denmark and became nationwide in May 2020. Volunteer citizens can sign up via a smartphone application (app) and are dispatched to either start CPR or retrieve a nearby automated external defibrillator (AED). The pilot study of the Danish citizen responder programme found that citizen responders arrived before the EMS in 42% of all OHCA. The arrival of a citizen responder before EMS was associated with a 3.73 odds for bystander defibrillation and 1.76 odds for bystander CPR [6]. Despite a wide implementation of citizen responder programmes in many countries, very little is known about the interaction and collaboration between the EMS personnel and citizen responders. A previous study found that citizen responders perceived the interaction with paramedics during OHCA as positive but that a minority of the ambulance personnel were perceived as having a negative attitude towards citizen responders [11]. In addition, citizen responder intervention could potentially be perceived as an obstructive element or interrupt a well-rehearsed high-performance teamwork between EMS personnel. Many citizen responders are lay persons who may not have been in an emergency situation previously. Therefore, the aim of this descriptive cross-sectional study was to investigate how emergency physicians perceive dispatched citizen responders’ participation in OHCA resuscitation.

## Method

### Study design and setting

In this cross-sectional study we investigated the collaboration between emergency physicians and citizen responders through a 23-item questionnaire sent to all emergency physicians in the Capital Region of Denmark. The region covers 2563 km^2^ and houses 1.847 million inhabitants of whom 40,279 (2181/100,000 inhabitants) were registered as citizen responders when the survey was conducted [12, 13]. The OHCA incidence in Denmark is 88/100,000 inhabitants, 74% of these occurs in private homes and in 2019 79.1% of OHCA patients received bystander CPR and 10.5% received bystander defibrillation [14]. The EMS in the Capital Region of Denmark consists of one emergency dispatch centre that activates a two-tiered response to all suspected OHCAs. The standard response includes the nearest ambulance and an emergency mobile critical care unit staffed with an emergency physician. Depending on availability and response time, first responders (fire crews) may also be dispatched. EMS management of OHCA is according to the ERC guidelines for BLS and ALS [15, 16]. In addition, the emergency dispatch centre can activate volunteer citizen responders. During the study, citizen responders were activated to all OHCAs except for traumatic cardiac arrests, children < 8 years, unsafe surroundings or when an AED was not indicated (e.g. at nursing homes where trained personnel is present). The emergency dispatch centre also instructs the present bystanders in initiating CPR (dispatch assisted CPR) and, when possible, encourages additional bystanders to retrieve the nearest on-site AED.

### The citizen responder Programme and the Danish AED network

Volunteer citizens over the age of 18 can register through the citizen responder app (Heartrunner) [13]. Training in CPR and AED use are highly recommended but not mandatory for registration. Yet, around 99% of the citizen responders report having received CPR training before registration and 26% reported being healthcare workers [6].

The citizen responder app is linked to the Danish AED network, containing approximately 6500 AEDs (137 AEDs/100,000 inhabitants/1000 km^2^) in the Capital Region of Denmark, 48.1% of these were accessible 24 h a day, 7 days a week (data accessed on October 2020) [17]. In case of a suspected OHCA, up to 20 citizen responders located within a radius of maximum 1.8 km are activated by the dispatch centre in parallel to the EMS. Four out of five citizen responders who accept the alarm are navigated to the scene via a nearby accessible AED using the accessibility information from the Danish AED Network. One out of five is directed straight to the OHCA to start CPR. The citizen responder programme has previously been described in detail [6].

### The questionnaire and study population

The questionnaire consisted of 23 sequential questions; 12 regarded the interaction and collaboration with citizen responders and the tasks they were given, four questions explored any potential obstructions in the interaction, one question investigated the perceived CPR quality provided by citizen responders, two questions regarding the physicians use of defusing, three questions were used to characterise the emergency physicians and one question left the possibility to suggest ways to optimise the programme, (see Additional file 1 for full questionnaire). The questionnaire was designed by four medical doctors working with the citizen responder programme, one of them being an emergency physician at the Copenhagen EMS. The questionnaire was validated for content and clarity using the cognitive interviewing technique interviewing five emergency physicians from the Copenhagen EMS and adjusted accordingly [18].

The questionnaire was sent to all 65 emergency physicians employed by the Capital Region of Denmark between June 9 and December 13, 2019. All emergency physicians included were medical doctors specialized in anaesthesiology. The questionnaire was constructed and sent out online through the web-based survey software ‘RedCap’ and distributed to the participants via e-mail [19]. In case of missing response, the emergency physicians were contacted personally and encouraged to answer the questionnaire. All participants agreed to publication of the results.

### Statistical analysis

Descriptive data were presented as medians with interquartile range and minimum and maximum values for continuous variables and frequencies and percentages for categorial data. A Chi-square test was used to analyse categorical data. Data analysis was performed using ‘RStudio’ version 3.6.

## Results

### Study population

All 65 emergency physicians working at the Copenhagen EMS in June 2019 received the questionnaire with an overall response rate of 87.7%(*n* = 57). The corresponding figure was 81.5%(*n* = 53) among emergency physicians who had previously engaged with citizen responders in OHCA resuscitation. The median age was 48 years (range 36–64), 78.9% were male, and the median time of experience as an emergency physician was 8 years (range 0.67–24). Of the 57 emergency physicians 93% (53/57) had interacted with a citizen responder at least once and 45.6% (26/57) had interacted with citizen responders more than ten times, Table 1. The non-responders (*n* = 8) included four females and four males, were slightly older than the study population but with no difference in working experience (Table 1).

**Table 1:** Characteristics of the Emergency Physicians

	*Study population*		*Non-responders*	
	(n = 57)		(n = 8)	
	*n*	*%*	n	%
**Sex**				
Male	45	78.9	4	50.0
Female	12	21.1	4	50.0
**Age**				
Median (IQR, Q1-Q3)	48.0 (45.0–55.3)		51.9 (50.0–58.3)	
**Time as an emergency physician (years)**				
Median (min:max)	8.0 (0.7: 24.0)		8.6 (4.8: 8.6)	
**Number of times the emergency physicians had interacted with a citizen responder**				
0	4	7.0		
1–4	12	21.1		
5–9	15	26.3		
> 10	26	45.6		

### Emergency physicians’ perception and collaboration with citizen responders

**Fig. 1:**
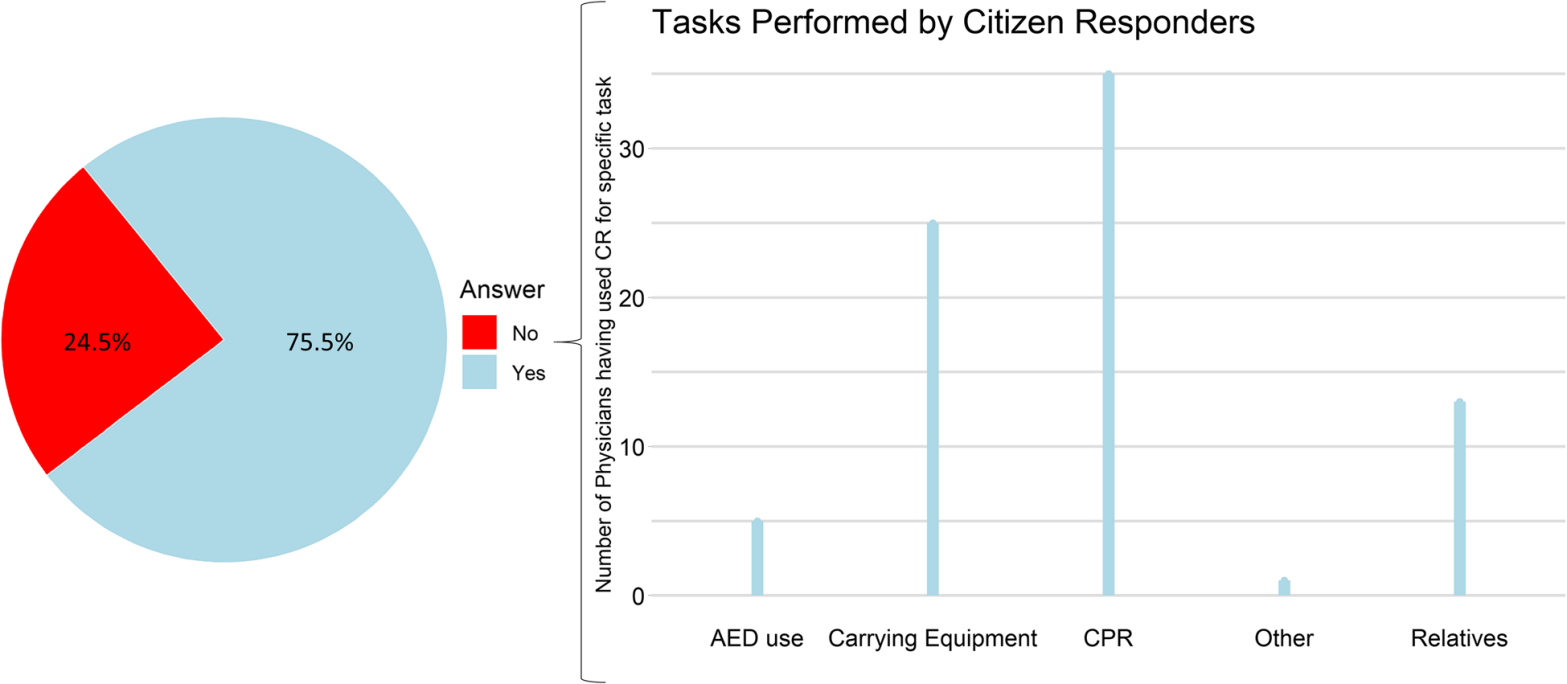
Assigned Tasks when the Citizen Responder Arrived Before EMS. The figure illustrates the percentage (%) of emergency physicians who continued to use the citizen responder’s help if the citizen responder was already at the out-of-hospital cardiac arrest (OHCA) scene upon their arrival (*n* = 53). It illustrates what tasks the emergency physicians assigned to the citizen responder; controlling the automated defibrillator (AED use), carrying equipment, continued chest compressions (CPR), ‘Other’ tasks (the only person answering “Other” explained this by “holding the infusion set”) or talking to relatives (Relatives). More than one option could be checked

**Fig. 2:**
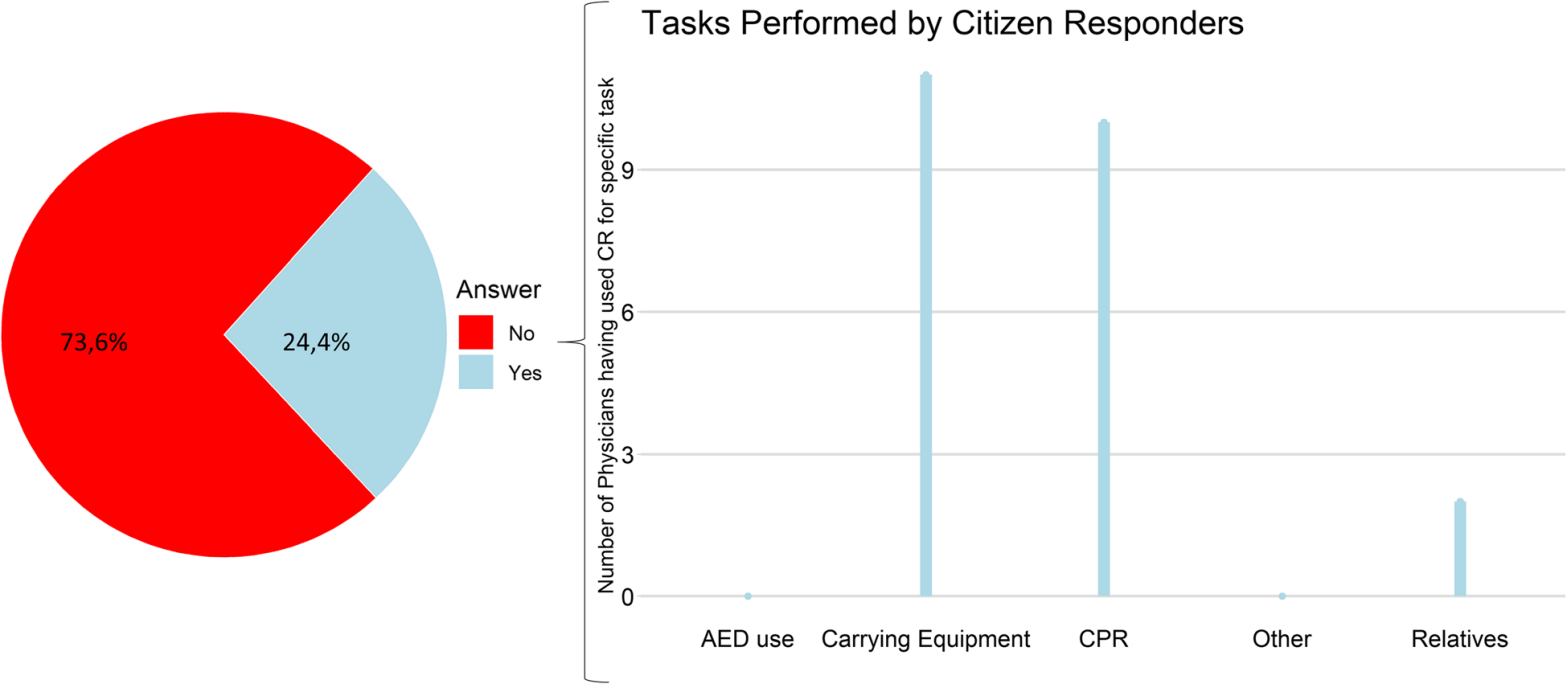
Assigned Tasks when the Citizen Responder Arrived After EMS. The figure illustrates the percentage (%) of emergency physicians who used citizen responders even when the citizen responder arrived at the out-of-hospital cardiac arrest (OHCA) location after themselves (n = 53). It illustrates what tasks emergency physicians assigned to the citizen responder; managing the automated defibrillator (AED use), carrying equipment, continued chest compressions (CPR), ‘Other’ tasks, or talking to relatives (Relatives). More than one option could be checked

Among the 53 emergency physicians who had collaborated with citizen responders, 92.5% considered it relevant to activate citizen responders to OHCA and 84.9% reported that citizen responders had the required basic skills to perform CPR. The collaboration was evaluated as ‘always helpful’ or ‘almost always helpful’ by 67.9% of the emergency physicians. Almost half (47.2%, 25/53) of the emergency physicians had defused a citizen responder after a resuscitation attempt. The emergency physicians used citizen responders’ help more often when the citizen responders were already at the scene before EMS arrival (75.5%, 40/53) compared with when the citizen responders arrived at the scene after EMS (26.4%, 14/53, *p* < 0.001). When arriving at the OHCA scene before the EMS, citizen responders were most often assigned to continue chest compressions (87.5% [35/40]), carrying equipment (62.5% [25/40]) and to take care of the relatives (32.5% [13/40]), Fig. 1. Citizen responders were assigned to the same tasks when arriving after EMS (continue chest compressions: 71.5%[10/14], carrying equipment: 78.5%[11/14] and taking care of relatives: 14.3%[2/14], Fig. 2).

Despite receiving the extra help from citizen responders, 79.2% (42/53) of the emergency physicians would still require an extra ambulance to complete similar tasks as those carried out by the citizen responders.

Citizen responders were also considered to be useful to guide EMS personnel to the patient and clearing the surroundings of the patient. A total of 20.8% of emergency physicians reported that citizen responders distracted their professional work because of physical or communicative barriers. Furthermore, 69.8% found it hard to differentiate between citizen responders and relatives at the scene. To prepare citizen responders for interacting with healthcare professionals, and thereby strengthen the teamwork, it was suggested that citizen responders are informed of EMS personnel’s tasks and working routines during a resuscitation attempt, and that they always present themselves clearly as citizen responders at the OHCA scene.

## Discussion

This cross-sectional study investigated the interaction between emergency physicians and citizen responders during OHCA resuscitation among all emergency physicians at Copenhagen EMS. Almost all (93%, *n* = 53/57) emergency physicians had interacted with a citizen responder and the majority (67.9%, *n* = 36/53) subjectively evaluated the collaboration as helpful. Most stated that citizen responders had the qualified skills to perform CPR and could be useful for tasks other than CPR, potentially even when arriving after the emergency physician. This study contributes with important insights about the collaboration between EMS personnel and citizen responders from the EMS’s perspective. It highlights that citizen responders can be a useful extra resource for the EMS besides providing CPR and using an AED.

App dispatch of volunteer citizens to OHCA resuscitation is currently developed and investigated in many countries to improve survival after OHCA [4, 7, 20]. A widespread of technologies and programmes exist; some are based on text messages others use smartphone applications, some dispatch citizen responders to private homes others only to public areas. The number of citizen responders alerted and the response radius also varies between programmes [4, 21]. The citizen responder programme in Denmark resembles the citizen responder programmes in several other European countries in many ways by using an app incorporated with a map using live AED locations to activate both laypersons and healthcare professionals [1, 4]. Many of the European citizen responder programmes dispatch citizen responders to private homes in contrast to the PulsePoint citizen responder programme in the USA where citizen responders are alerted to OHCAs in public locations [22]. Despite the many different technologies being used, the most optimal system setting to improve survival after OHCA is unknown.

The citizen responder programme in Denmark is one of few where CPR training is not mandatory [1, 4]. Our study only investigated emergency physicians’ perspective of the citizen responders and their CPR skills and not the objective CPR quality according to ERC guidelines [15]. However, we found that 67.9% of emergency physicians perceived citizen responders as helpful and 84.9% of emergency physicians subjectively assessed the quality of CPR provided by citizen responders as satisfactory. This assessment was underlined since many of the emergency physicians asked citizen responders to continue CPR even after EMS had arrived. Some physicians even assigned citizen responders to perform CPR when they arrived after the EMS. Having CPR training is highly recommended but not mandatory when signing up as a citizen responder in Denmark. However 99% of the citizen responders report previous CPR training before registration [6]. Studies using quantitative data from AEDs to determine the quality of bystander CPR conclude that bystander CPR quality often meets the European Resuscitation Council guidelines, [23, 24]. Opposed to random bystanders, citizen responders have chosen to register and assist with CPR in case of cardiac arrest and are therefore more mentally prepared and could be assumed to be better than random bystanders in performing CPR.

A citizen responder programme in France, StayingAlive, was evaluated by Derkenne et al. and found an association between activation of CPR trained volunteers and increased OHCA survival [25]. Originally, the programme included trained volunteers, but in 2019 the programme expanded to accept non-trained volunteers [25, 26]. Volunteers not trained in CPR can be used as a resource for non-CPR tasks, for example bringing the nearest AED to the cardiac arrest location. Accordingly, our study found that citizen responders could help with non-CPR related tasks such as taking care of relatives, directing the EMS to the arrest location, and carrying equipment for the EMS crew. Non-CPR tasks, as described in this study, are not in the standard curriculum of a basic life support course. Expanding the potential use of citizen responders, especially regarding the care of relatives, might be a useful source of help in an OHCA setting. Therefore, implementing psychological first aid and information about EMS working structures as part of basic life support training might help to prepare and optimise the role of citizen responders. Instructions in non-CPR tasks could also be implemented in the responder app in the same way video instruction on performing CPR, using an AED, supporting relatives and how to react after a resuscitation attempt are already accessible in the app.

Both the study by Derkenne et al. and our study found that EMS personnel continued to use the citizen responders’ help, even when they arrived after the EMS. This emphasizes that citizen responders can play an important role in treating OHCA patients by supporting the EMS with extra hands in situations with sparse resources. However, the continued use of citizen responders in a resuscitation attempt could deviate from the “pitstop approach to CPR” mentioned by the Global Resuscitation Alliance Ten Programs of how to increase survival in OHCA, Program 3 [27]. Citizen responders do not have the same prerequisites to contribute to the team performance as the professional EMS personnel do. Therefore, the interaction could be perceived as an obstruction by EMS personnel, which was reported by 20.8% of the emergency physicians in this study. Informing citizen responders of the pitstop approach to high quality CPR and tasks of the EMS could increase the teamwork, as suggested by the emergency physicians. Importantly, teamwork has been reported to facilitate bystander resuscitation efforts [28]. Thus, including teamwork training in the basic life support courses could be helpful to improve citizen responder performance during resuscitation attempt. The Global Resuscitation Alliance, the European Resuscitation Council, and American Heart Association (AHA) guidelines 2020 all encourage the use app dispatched citizen responders to improve early CPR and defibrillation [27, 29, 30]. Collaboration between the professional EMS and citizen responders will therefore often occur in many communities in the future. An improvement of this collaboration and the transitional time between early CPR and high-performance CPR could optimise the role of citizen responders in OHCA resuscitation.

### Study limitations

The survey was not completed after each interaction with a citizen responder and thus potential recall bias could therefore occur in this study. The reason why non-responders did not answer the survey were unexplored and thus unknown to the authors. Non-responders were more often female and older than the responders, but they had just as much working experience as the study population. Sex and age were not considered confounders which is why this difference was not expected to impact the outcome. The study only included emergency physicians and did not include ambulance personnel who are also part of the professional EMS response in the Capital Region of Denmark. The full description of citizen responders’ collaboration with EMS personnel is therefore beyond the scope of this study. Since the ambulance personnel often arrives at the scene before emergency physicians, citizen responders might provide an even bigger resource assuming that extra hands are more valuable when only one ambulance with two EMS personnel are present. Additionally, the Capital Region of Denmark has short EMS response times compared with many other regions which might influence the interaction and use of citizen responders by EMS personnel during OHCA resuscitation. Accordingly, the EMS personnel’s perception of the interaction with citizen responders might differ in regions with longer EMS response times and more rural areas [6].

Not all emergency physicians had defused a citizen responder, but the HeartRunner programme offers defusing to all citizen responders. Furthermore, a recent study shows that 4 weeks after participating in a resuscitation attempt 86% of citizen responders had low perceived stress and 1%(1/102) had sought professional help due to psychological impact. Moderate perceived stress (14%) was significantly associated with specific personal traits such as neuroticism and openness to experience [31]. These results can possibly help identify citizen responders in the risk of psychological distress.

This study was limited to describe the EMS personnel’s perception and assessment of the interaction between EMS personnel and citizen responders. Furthermore, observations regarding the CPR quality provided by the citizen responders is based on subjective assessments by EMS personnel.

## Conclusion

Besides providing CPR and using an AED, citizen responders can play an important role in treating OHCA patients by supporting the EMS with extra hands in situations with sparse resources. Citizen responders were also considered an extra helpful resource for non-CPR related tasks during resuscitation such as taking care of relatives. To optimize the role of citizen responders, information on the EMS working structure and instructions in psychological first aid might be considered to citizen responders before registration.

## Supplementary Information

**Additional file 1.** The full survey.

**Additional file 2.** The full dataset.

## Data Availability

Full survey and dataset are available for the public, see “Additional Files”.
