# Supplementary material for: Collaboration between emergency physicians and citizen responders in out-of-hospital cardiac arrest resuscitation
Source: Scand J Trauma Resusc Emerg Med. 2021 Aug 3;29:110. doi: 10.1186/s13049-021-00927-w (PMC8330065; doi:10.1186/s13049-021-00927-w)
Supplement: Supplementary file 1 — Additional file 1. The full survey. [file 13049_2021_927_MOESM1_ESM.pdf]

Appendix 1

Survey:

Survey sent to all the emergency physicians working in the Capital Region of Denmark in the time period June 9th, 2019 and December 13th, 2019. The original survey is in Danish. This is the English translation.

1. **Have you met a citizen responder at a cardiac arrest?**
   - Yes
   - No (go to 14.)
2. **How many times have you interacted with a citizen responder?**
   - 1-4 times
   - 5-9 times
   - 10 times or more
3. **Do you find it relevant that citizen responders are activated for assumed cardiac arrests?**
   - Yes
   - No
4. **Do you consider the presence of the citizen responder as a help (By help is meant help in any possible way, both considering practical tasks, the CPR itself, support for the relatives and so on)?**
   - Always
   - Almost always
   - Neither nor
   - Rarely
   - Never
5. **If the citizen responder is at the OHCA scene BEFORE you, do you then use their help after your arrival?**
   - Yes (go to 5.1.)
   - No (go to 6.)

   5.1. What did the citizen responder help with?
   - Carrying down the equipment (go to 6.)
   - Continued chest compression (go to 6.)
   - For defibrillation with the AED (go to 6.)
   - Talking with relatives (go to 6.)
   - Other (go to 5.2.)

   5.2 . What does "other" involve?
   - __________ (go to 6.)
6. **If the citizen responder is at the OHCA scene AFTER you, do you then use their help after your arrival?**
   - Yes (go to 6.1.)
   - No (go to 7.)

   6.1. What did the citizen responder help with?
   - Carrying down the equipment (go to 7.)

- Continued chest compression (go to 7.)
- For defibrillation with the AED (go to 7.)
- Talking with relatives (go to 7.)
- Other (go to 6.2.)

6.2 What does “other” involve?

- __________ (go to 7.)

7. **If there are one or more citizen responders at the OHCA scene, will you then be prone to not acquire an extra resource/ambulance to carrying equipment and so on?**
   - Yes
   - No
8. **Do you think the citizen responder improves the access to the patient, e.g. by showing the way or clearing the space surrounding the patient?**
   - Yes
   - No
9. **Is it your experience that the citizen responder can be obstructive for optimal work routines and workflow?**
   - Yes (go to 12.)
   - No

9.1. In what way have you experienced the citizen responder has been obstructive?

- Communication with the Citizen responder removes focus from ALS (go to 10.)
- The Citizen responder was a physically in the way. (go to 10.)
- Other (go to 9.2.)

9.2. What does “other” involve?

- __________ (go to 10.)

10. **Is it your impression that the citizen responders have the necessary basic CPR qualities?**
    - Yes
    - No
11. **Do you find it hard to differentiate between the citizen responder and the relatives?**
    - Yes
    - No
12. **Have you experienced the need for aggregation/defusing of the citizen responders after an assignment?**
    - Yes
    - No
13. **Have you taken the initiative to or taken part of such a defusing of the citizen responder immediately after the end of the assignment?**
    - Yes
    - No
14. **For how long have you been working as an emergency physician?**
    - ______
15. **Your age**
    - ______
16. **Your sex**
    - Male
    - Female

17. Have you any suggestions on how to prepare the citizen responders in order to obtain the best possible teamwork with the emergency physicians?

- ○ ____
