# Supplementary material for: Collaboration between emergency physicians and citizen responders in out-of-hospital cardiac arrest resuscitation
Source: Scand J Trauma Resusc Emerg Med. 2021 Aug 3;29:110. doi: 10.1186/s13049-021-00927-w (PMC8330065; doi:10.1186/s13049-021-00927-w)
Supplement: Supplementary file 2 — Additional file 2. The full dataset. [file 13049_2021_927_MOESM2_ESM.pdf]

Appendix 2

Full Dataset

		N	%
1	Have you met a citizen responder at a cardiac arrest?	Total count (N)	57	
		Yes	53	92,98
		No	4	7,02
2	How many times have you interacted with a citizen responder?	Total count (N)	53	
		1-4 times	12	22,64
		5-9 times	15	28,30
		10 times or more	26	49,06
3	Do you find it relevant that citizen responders are activated for assumed cardiac arrests?	Total count (N)	53	
		Yes	49	92,45
		No	4	7,55
4	Do you consider the presence of the citizen responder as a help (By help is meant help in any possible way, both considering practical tasks, the CPR itself, support for the relatives and so on)?	Total count (N)	53	
		Always	5	9,43
		Almost always	31	58,49
		Neither nor	12	22,64
		Rarely	5	9,43
		Never	0	0,00
5	If the citizen responder is at the OHCA scene BEFORE you, do you then use their help after your arrival?	Total count (N)	53	
		Yes	40	75,47
		No	13	24,53
5.1	What did the citizen responder help with? (check one or more boxes)	Total count (N)	40	
		Carrying equipment	25	62,50
		Continued Chest Compressions	35	87,50
		Defibrillation with AED	5	12,50
		Talking to Relatives	13	32,50
		Other	1	2,50
5.2	What does "other" involve?	Total count (N)	1	
		Holding the infusion set	1	1,00

6	**If the citizen responder is at the OHCA scene AFTER you, do you then use their help after your arrival?**	Total count (N)	53	
		Yes	14	26,42
		No	39	73,58
6.1	**What did the citizen responder help with? (check one or more boxes)**	Total count (N)	14	
		Carrying equipment	11	78,57
		Continued Chest Compressions	10	71,43
		Defibrillation with AED	0	0,00
		Talking to Relatives	2	14,29
		Other	0	0,00
6.2	**What does "other" involve?**	Total count (N)	0	0,00
7	**If there are one or more citizen responders at the OHCA scene, will you then be prone to not acquire an extra resource/ambulance e.g. to carrying equipment ?**	Total count (N)	53	
		Yes	11	20,75
		No	42	79,25
8	**Do you think the citizen responder improves the access to the patient, e.g. by showing the way or clearing the space surrounding the patient?**	Total count (N)	53	
		Yes	35	66,04
		No	18	33,96
9	**Is it your experience that the citizen responder can be obstructive for optimal work routines and workflow?**	Total count (N)	53	
		Yes	11	20,75
		No	42	79,25
9.1	**In what way have you experienced the citizen responder has been obstructive?**	Total count (N)	11	
		The citizen responder was physically in the way	6	54,55
		Communication with the citizen responder removes focus from ALS	5	45,45
		Other	3	27,27
9.2	**What does "other" involve?**	Total count (N)	3	
		"Had endned the treatment by him/her self"	1	33,33
		"Interfering with the treatment, especially if they are doctors without insight in the prehospital field"	1	33,33
		"Too many came"	1	33,33

10	**Is it your impression that the citizen responders have the necessary basic CPR qualities?**	Total count (N)	53	
		Yes	45	84,91
		No	8	15,09
11	**Do you find it hard to differentiate between the citizen responder and the relatives?**	Total count (N)	53	
		Yes	37	69,81
		No	16	30,19
12	**Have you experienced the need for aggregation/defusing of the citizen responders after an assignment?**	Total count (N)	53	
		Yes	20	37,74
		No	33	62,26
13	**Have you taken the initiative to or taken part of such a defusing of the citizen responder immediately after the end of the assignment?**	Total count (N)	53	
		Yes	25	47,17
		No	28	52,83
14	**For how long have you been working as an emergency physician?**	Total count (N)	57	
		0.67 years (8 months)	1	1,75
		1 year	1	1,75
		2 years	1	1,75
		3 years	2	3,51
		5 years	5	8,77
		6 years	10	17,54
		7 years	3	5,26
		8 years	6	10,53
		9 years	3	5,26
		10 years	4	7,02
		>10 year	2	3,51
		11 years	1	1,75
		12 years	3	5,26
		13 years	1	1,75
		14 years	3	5,26
		15 years	3	5,26
		16-17 years	1	1,75
		17 years	1	1,75
		18 years	1	1,75
		19 years	2	3,51
		20 years	2	3,51
		24 years	1	1,75

15	**Your age**	Total count (N)	56	
		36	1	1,79
		39	1	1,79
		41	2	3,57
		42	1	1,79
		43	3	5,36
		44	1	1,79
		45	7	12,50
		46	2	3,57
		47	5	8,93
		48	6	10,71
		49	5	8,93
		50	1	1,79
		51	1	1,79
		53	2	3,57
		54	3	5,36
		55	1	1,79
		56	1	1,79
		57	1	1,79
		58	1	1,79
		59	3	5,36
		60	1	1,79
		61	3	5,36
		62	1	1,79
		63	1	1,79
		64	2	3,57
16	**Your sex**	Total count (N)	57	
		Male	45	78,95
		Female	12	21,05
17	**Have you any suggestions on how to prepare the citizen responders in order to obtain the best possible teamwork with the emergency physicians?**	Total count	17	
		No	2	3,51
		They should be marked e.g. with a west. Could you for instance throw some information nights where they could hear how we work and what we are going to go when we arrive at the scene.	1	1,75
		optimal CPR, taking care of the relatives	1	1,75

	"I have answered yes/no where I would have answered "sometimes".	1	1,75
	The skill to shortly summarize the situation if they arrive before the PA/ALB	1	1,75
	Skip them in the Capital Region, probably fine in the rest of the country	1	1,75
	E-learning they shall pass before becoming a citizen responder. I'm meeting many incompetent citizen responders that are not ready to see dead people.	1	1,75
	Marking with a west or ID card	2	3,51
	information meetings / training for citizen responders	3	5,26
	They actively orally present themselves as citizen responders	1	1,75
	Make sure that all citizen responders are sufficient educated in CPR	1	1,75
	The problem with an otherwise great idea is that too many citizen responders arrive, it is confusing instead of being a resource	1	1,75
	Inform the citizen responders to avoid rescue breaths doing CPR due to risk of infection, instead focus on CPR and AED use.	1	1,75

Non-responders, n = 8		n	%
**Sex**			
	Male	4	50,0
	Female	4	50,0
**Age**			
	60,8	1	12,5
	50,9	1	12,5
	63,8	1	12,5
	50,8	1	12,5
	47,1	1	12,5
	52,8	1	12,5
	57,4	1	12,5
	47,6	1	12,5
**Seniority as an emergency physician (years)**			
	8,6	5	62,5
	8,3	1	12,5
	4,8	1	12,5
	5,9	1	12,5
